# Dynamic interaction of SARAF with STIM1 and Orai1 to modulate store-operated calcium entry

**DOI:** 10.1038/srep24452

**Published:** 2016-04-12

**Authors:** Letizia Albarran, Jose J. Lopez, Nidhal Ben Amor, Francisco E. Martin-Cano, Alejandro Berna-Erro, Tarik Smani, Gines M. Salido, Juan A. Rosado

**Affiliations:** 1 Department of Physiology (Cellular Physiology and Muscle Physiology Research Groups), University of Extremadura, 10003 Cáceres, Spain; 2 Department of Medical Physiology and Biophysics, Institute of Biomedicine of Sevilla, Sevilla, Spain

## Abstract

Ca^2+^ influx by store-operated Ca^2+^ channels is a major mechanism for intracellular Ca^2+^ homeostasis and cellular function. Here we present evidence for the dynamic interaction between the SOCE-associated regulatory factor (SARAF), STIM1 and Orai1. SARAF overexpression attenuated SOCE and the STIM1-Orai1 interaction in cells endogenously expressing STIM1 and Orai1 while RNAi-mediated SARAF silencing induced opposite effects. SARAF impaired the association between Orai1 and the Orai1-activating small fragment of STIM1 co-expressed in the STIM1-deficient NG115-401L cells. Cell treatment with thapsigargin or physiological agonists results in direct association of SARAF with Orai1. STIM1-independent interaction of SARAF with Orai1 leads to activation of this channel. In cells endogenously expressing STIM1 and Orai1, Ca^2+^ store depletion leads to dissociation of SARAF with STIM1 approximately 30s after treatment with thapsigargin, which paralleled the increase in SARAF-Orai1 interaction, followed by reinteraction with STIM1 and dissociation from Orai1. Co-expression of SARAF and either Orai1 or various N-terminal deletion Orai1 mutants did not alter SARAF-Orai1 interaction; however, expression of C-terminal deletion Orai1 mutants or blockade of the C-terminus of Orai1 impair the interaction with SARAF. These observations suggest that SARAF exerts an initial positive role in the activation of SOCE followed by the facilitation of SCDI of Orai1.

Store operated Ca^2+^ entry (SOCE) is a major pathway of calcium influx in non-excitable cells, and is essential for the activation of many cellular processes. This mechanism is initiated by the depletion of the intracellular Ca^2+^ stores, mainly the endoplasmic reticulum (ER). The two major components of SOCE are STIM1^1^,^2^, the Ca^2+^ sensor of the ER that communicates the signal of store depletion to the plasma membrane resident Ca^2+^ channel, and the channel pore-forming Orai1^3^,^4^,^5^.

Orai1 is a highly Ca^2+^-selective, inward-rectifying, channel that has been reported to be regulated by Ca^2+^ in two possible ways named fast Ca^2+^-dependent inactivation (FCDI), which occurs within milliseconds^6^,^7^, and slow Ca^2+^-dependent inactivation (SCDI) that inactivates Orai1 over 2–3 min to prevent Ca^2+^ overload^8^,^9^. The later mechanism is activated by Ca^2+^ entering through Orai1 channels, and constitutes a slow negative feedback process. SCDI has been reported to include both store refilling-dependent and independent components in Jurkat T cells^8^; although it has been found to be entirely independent on store refilling in rat basophilic leukemia cells^9^.

STIM1 N-terminus exhibits an EF-hand motif, which, upon Ca^2+^ dissociation, leads to oligomerization and clustering of STIM1 into puncta located at the ER–plasma membrane junctions^10^. This transition is accompanied by a conformational reorganization of its cytosolic region from a closed to an extended state leading to the exposition of the SOAR domain (amino acids 344–442^11^; also known as OASF (233–450/474)^12^, CAD (342–448)^13^ or Ccb9 (339–444)^14^), which results in full activation of Orai1^15^. The crystal structure of SOAR has revealed that this domain exists as a V-shaped dimer^16^. In the resting state, STIM1 may exist mostly as dimers with the SOAR dimer likely occluded. In the inactive state, the Ca^2+^ bound intraluminal region remains monomeric^10^ but, upon Ca^2+^ store depletion, the N-terminal region of STIM1 releases Ca^2+^ and oligomerizes resulting in the conformational change that releases the SOAR domain^16^.

Recent studies have identified a C-terminal inhibitory domain (CTID; amino acids 448–530) downstream of the SOAR domain, whose deletion leads to spontaneous clustering of STIM1 and activation of Orai1 in the absence of Ca^2+^ store depletion^17^. CTID has been reported to mediate the interaction of STIM1 with a new regulatory protein named SARAF^17^. SARAF is a 339-amino acid long protein with a putative single transmembrane domain and C-terminal serine/proline and arginine rich regions. SARAF is located in the membrane of the endoplasmic reticulum and interacts with the CTID region of STIM1 to mediate SCDI of Orai1 channels^18^. CTID has been described to have two functional lobes, STIM1(448–490) and STIM1(490–530), which cooperate to modulate the access of SARAF to the SOAR region, so that in the resting state, when the intracellular stores are filled with Ca^2+^, the CTID lobes facilitate access of SARAF to SOAR to keep SOAR in an inactive state, while store depletion results in an initial dissociation of SARAF, to allow activation of STIM1 and SOCE, followed by a reinteraction of SARAF with SOAR, facilitated by the STIM1(490–530) lobe, a process that has been associated with the SCDI of Orai1^17^.

In the present study we have investigated the interaction of SARAF with STIM1 and Orai1 during the activation of SOCE. Our results indicate that SARAF is a modulator of the interaction between STIM1 and Orai1 channels that, during the initial steps of the activation of SOCE, transiently dissociates from STIM1 to associate with the C-terminus of Orai1 to activate Ca^2+^ entry, and then reinteracts with STIM1 to mediate SCDI. These findings shed new light on the regulatory mechanism of SOCE.

## Results

### SARAF regulates store-operated Ca^2+^ entry and STIM1-Orai1 interaction

**Figure 1:**
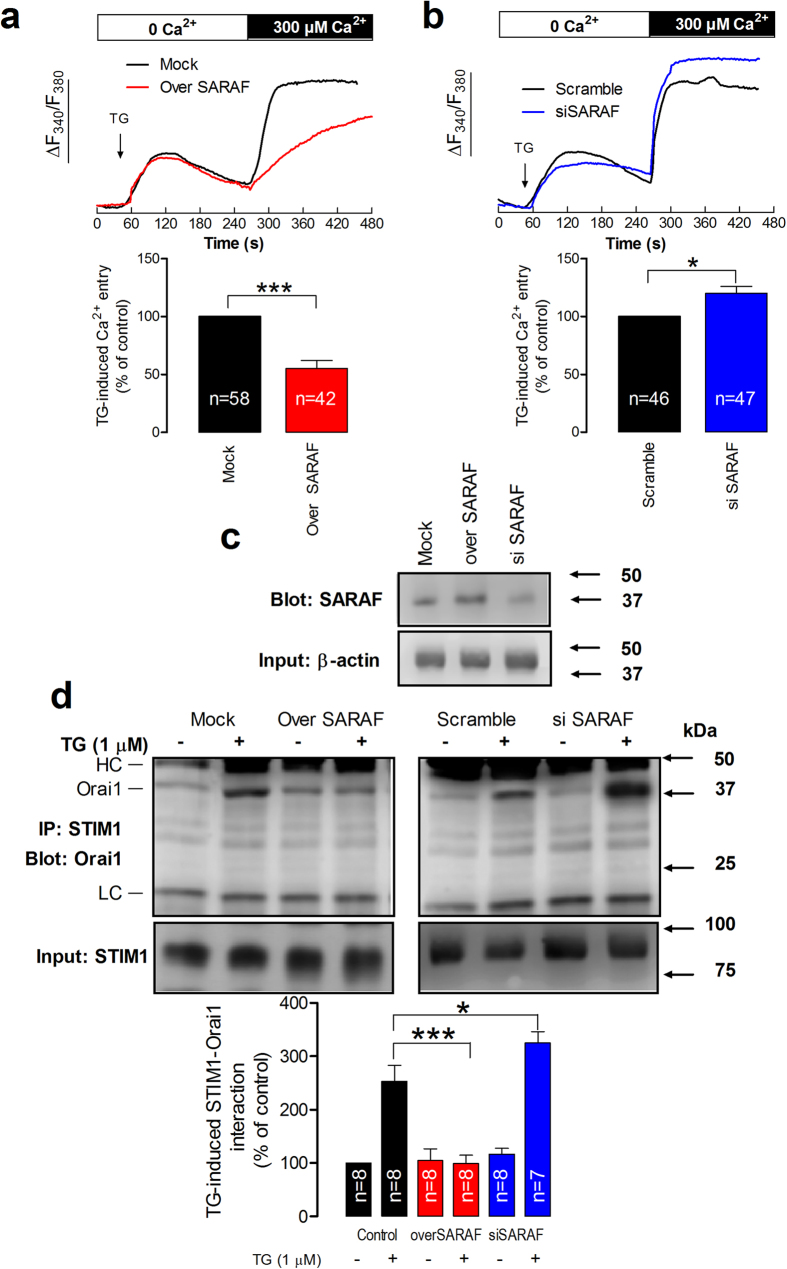
SARAF regulates TG-induced SOCE and STIM1-Orai1 interaction. (**a,b**) MEG-01 cells overexpressing SARAF and mock-treated cells (**a**) or MEG-01 cells transfected with si SARAF or scramble plasmid (**b**) were perfused with a Ca^2+^-free medium (100 μM EGTA added) and then stimulated with TG (1 μM) followed by reintroduction of external Ca^2+^ (final concentration 300 μM) to initiate Ca^2+^ entry. Data are original traces representative of 42-58 experiments. Values are expressed as described in methods. The bar graph represents TG-induced Ca^2+^ entry as mean ± SEM and data are presented as percentage of control. (**c**) Cells overexpressing SARAF, treated with siSARAF and mock-treated cells were lysed and subjected to Western blotting with anti-SARAF antibody, followed by reprobing with anti-actin antibody for protein loading control. (**d**) MEG-01 cells overexpressing SARAF or transfected with si SARAF and their respective controls were stimulated with TG (1 μM) in a Ca^2+^-free medium (100 μM EGTA added) and three min later lysed. Whole cell lysates were immunoprecipitated (IP) with anti-STIM1 antibody and immunoprecipitates were subjected to 10% SDS-PAGE and subsequent Western blotting with a specific anti-Orai1 (aa 288–301 (Sigma)) antibody. Membranes were reprobed with the antibody used for immunoprecipitation for protein loading control. The panels show results from one experiment representative of 6–7 others. Molecular masses indicated on the right were determined using molecular-mass markers run in the same gel. HC, heavy chain of the antibody used for immunoprecipitation. The bar graph represents the quantification of STIM1-Orai1 association in resting and TG-treated cells. Results are recorded as arbitrary optical density units, expressed as mean ± S.E.M. and presented as percentage of control. * and *** represent p < 0.05 and p < 0.001, as compared to their respective controls.

As depicted in Fig. 1a, treatment of MEG-01 cells with thapsigargin (TG) in a Ca^2+^-free medium resulted in a transient rise in cytosolic Ca^2+^ concentration ([Ca^2+^]_c_) due to passive Ca^2+^ release from the intracellular stores. The subsequent SOCE, upon readdition of Ca^2+^ to the medium, was significantly inhibited by 45% in cells overexpressing SARAF (p < 0.001). As expected, SOCE was significantly enhanced by 20% in cells where endogenous SARAF levels were reduced by siRNA (Fig. 1b; p < 0.05).

To further explore the mechanism underlying the regulation of SOCE by SARAF we modified the expression of SARAF in MEG-01 cells endogenously expressing STIM1 and Orai1^19^, as well as SARAF (Fig. 1c), and investigated the role of SARAF in the interaction between STIM1 and Orai1. The experiments were performed in resting cells and upon stimulation with TG by looking for co-immunoprecipitation in whole cell lysates from cells overexpressing SARAF and SARAF-silenced cells. As shown in Fig. 1d, a detectable interaction between STIM1 and Orai1 was noticeable in resting cells, as previously described^19^. This interaction was significantly enhanced to 253 ± 30% of control after Ca^2+^ store depletion. Overexpression of SARAF abolished TG-evoked interaction between STIM1 and Orai1 without having any significant effect on the resting interaction between both proteins. Consistent with this, SARAF silencing significantly enhanced the STIM1-Orai1 interaction induced by TG to 325 ± 21% of control (p < 0.05) and had a negligible effect on this interaction in resting cells. Analysis of the specificity of the anti-Orai1 and anti-STIM1 antibodies is depicted in the supplementary Fig. 1.

**Figure 2:**
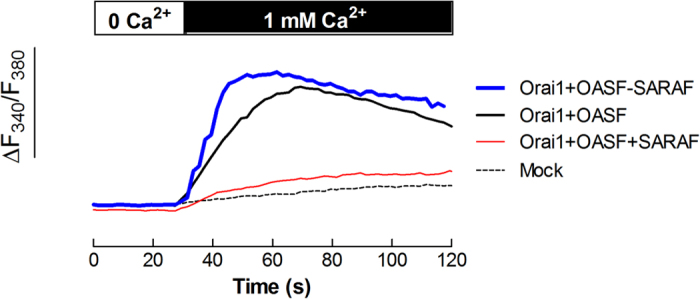
SARAF impairs OASF-dependent Orai1 function. NG115-401L cells were transfected with pEYPF-Orai1 and pEYPF-OASF in the absence of presence of SARAF or siSARAF or were mock treated, as indicated. After 48h cells were perfused with a Ca^2+^-free medium (100 μM EGTA added) followed by reintroduction of external Ca^2+^ (final concentration 1 mM) to initiate constitutive Ca^2+^ entry. Data are original traces representative of 42–58 experiments. Values are expressed as described in methods.

In an attempt to ascertain the role of SARAF in the STIM1-Orai1 association in Ca^2+^ store-depleted cells, we analyzed the interaction between Orai1 and the Orai1-activating small fragment (OASF; amino acids 233–474) of STIM1 in NG115-401L cells, which express a negligible amount of endogenous STIM1^20^,^21^. Cells were transfected with expression plasmids for pEYFP-Orai1 and pEYFP-OASF in combination with siSARAF or expression plasmid for SARAF. As shown in Fig. 2, co-expression of Orai1 and OASF resulted in a robust activation of Ca^2+^ entry independently of Ca^2+^ store depletion as compared to mock-treated cells, which did not display constitutive Ca^2+^ entry. Ca^2+^ entry induced by co-expression of Orai1 and OASF was impaired by overexpression of SARAF and enhanced by SARAF silencing (Fig. 2), thus suggesting that SARAF directly interferes with the interaction between the OASF region of STIM1 and Orai1.

### SARAF interacts with STIM1 and Orai1

**Figure 3:**
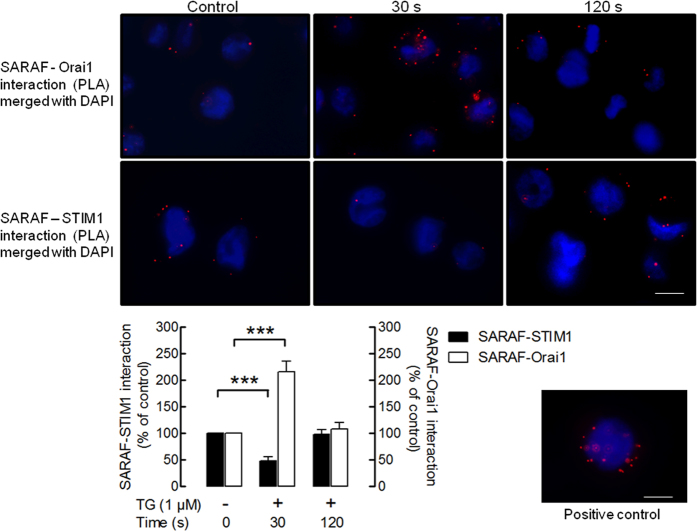
Dynamic interaction of SARAF with Orai1 and STIM1. MEG-01 cells were suspended in HBS containing 300 μM CaCl_2_ and were left untreated or stimulated with TG (1 μM) for 30 or 120 s. The cells were then fixed with 4% ice-cold paraformaldehyde and blocked using blocking solution from the Duolink *In Situ* Red kit^®^ from Sigma. Samples were incubated with primary antibodies: rabbit anti-SARAF and mouse anti-STIM1 or mouse anti-Orai1 (SAB4200273, Sigma) antibodies. A Duolink assay was subsequently performed according to the manufacturer’s instructions as described in Material and Methods. After performing the PLA procedure, images were taken with an Eclipse TE300 fluorescence microscope. Red spots represent individual dimers. The positive control is shown in the lower right corner. The bars indicate 10 μm. The bar graphs indicate association between SARAF and STIM1 or Orai1 expressed as the mean ± S.E.M. of 100–200 cells. The quantification of PLA signals was performed using ImageJ software. ***p < 0.001.

It has been suggested that SARAF is associated with STIM1 in resting cells and upon store depletion undergoes dissociation of STIM1 followed by reinteraction to activate SCDI^17^; however, the functional role of SARAF when it is dissociated from STIM1 remains unknown. We have now further explored the temporal relationship of the association of SARAF with STIM1 and Orai1 in MEG-01 cells. We have explored the interaction of SARAF with STIM1 or Orai1 by *in situ* proximity ligation assay (PLA). Resting MEG-01 cells or cells stimulated with TG (1 μM) for 30 or 120 s were subjected to PLA. As shown in Fig. 3 under resting conditions SARAF-Orai1 and SARAF-STIM1 interactions were detected in most cells analyzed. Treatment of MEG01 cells with TG (1 μM) for 30 s enhanced the interaction SARAF-Orai1 as detected by the increased number of red dots, as compared to control. However, the number of associations between SARAF and STIM1 per cell was significantly reduced (and we were unable to detected them in a number of cells) upon stimulation for 30 s with TG. After 120 s of treatment with TG the number of red dots/cell representative of SARAF-Orai1 as well as SARAF-STIM1 interaction returned to levels similar to those observed in resting cells. The quantification of the PLA signal for the interaction between SARAF and STIM1 or SARAF and Orai1 revealed that treatment with TG enhanced the interaction between SARAF and Orai1 reaching a maximum of 215 ± 21% of control (resting cells) after 30 s of stimulation and then returned to approximately the resting level after 120 s of treatment (Fig. 3; p < 0.001); Furthermore, TG significantly reduced the association between SARAF and STIM1 reaching a minimum of 48 ± 7% of control after 30 s of stimulated and then increased, reaching the resting level at 120 s of treatment (Fig. 3; p < 0.001). These findings support the dynamic interaction of SARAF with STIM1 and Orai1 upon treatment with TG.

**Figure 4:**
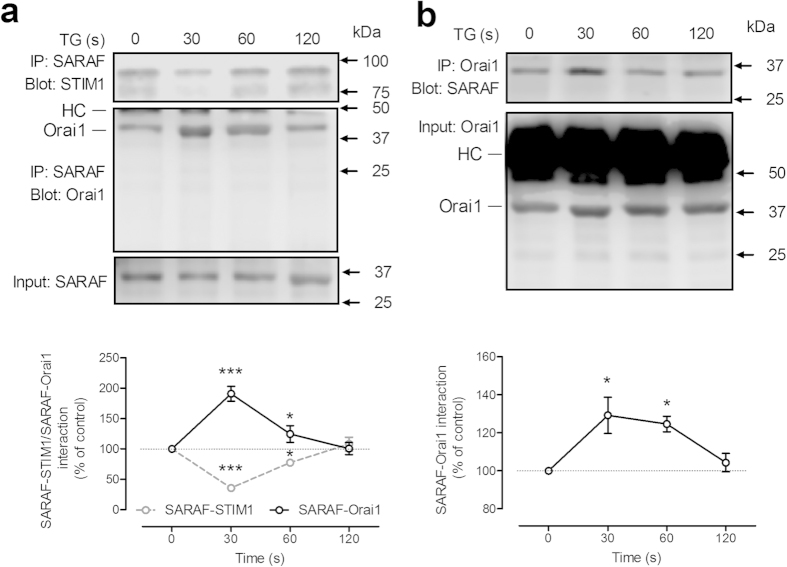
Time-course of TG-induced SARAF-STIM1 and SARAF-Orai1 co-immunoprecipitation. MEG-01 cells (**a**) and NG115-401L cells (**b**) were stimulated with TG (1 μM) in the presence of 300 μM extracellular Ca^2+^. Samples were removed 30s before the addition of TG and 30, 60 and 120 s after the treatment with TG. Whole cell lysates were immunoprecipitated (IP) with the indicated antibody and immunoprecipitates were subjected to 10% SDS-PAGE and subsequent Western blotting with specific anti-Orai1 (aa 288–301 (Sigma)), anti-SARAF and anti-STIM1 antibodies. Membranes were reprobed with the antibody used for immunoprecipitation for protein loading control. The panels show results from one experiment representative of 5 others. Molecular masses indicated on the right were determined using molecular-mass markers run in the same gel. HC, heavy chain of the antibody used for immunoprecipitation. Data represent the quantification of SARAF-Orai1 and SARAF-STIM1 association in resting and TG-treated cells. Results are recorded as arbitrary optical density units, expressed as mean ± S.E.M. and presented as percentage of control (resting cells). * and *** represent p < 0.05 and p < 0.001, as compared to controls.

We further explored the interaction between SARAF and both STIM1 and Orai1 by co-immunoprecipitation. As shown in Fig. 4a, a detectable interaction between SARAF and both STIM1 and Orai1 was appreciable in resting cells. Treatment of MEG-01 cells with TG evoked a time-dependent effect on the association of SARAF with STIM1 and Orai1. TG induced an initial significant decrease in SARAF-STIM1 interaction that reached a minimum of 35% of the resting level 30s after stimulation (p < 0.001). SARAF-STIM1 interaction then increased, exceeding basal levels approximately 120s after stimulation. In parallel with these findings, treatment with TG significantly increased the association between SARAF and Orai1 reaching a maximum of 190% of the resting level 30 s after its addition (p < 0.001) and then returned to the basal level at 120 s after stimulation (Fig. 4a).

In order to explore whether the interaction SARAF-Orai1 requires STIM1 we repeated the study in NG115-401L cells lacking STIM1. As reported in Fig. 4b, our results indicate that, in the absence of STIM1, TG evokes association between SARAF and Orai1, which reached a maximum 30 s after cell stimulation (p < 0.001). These findings indicate that STIM1 is not responsible for the dissociation of the interaction between SARAF and Orai1.

### SARAF modulates Ca^2+^ entry through Orai1 independently of STIM1

**Figure 5:**
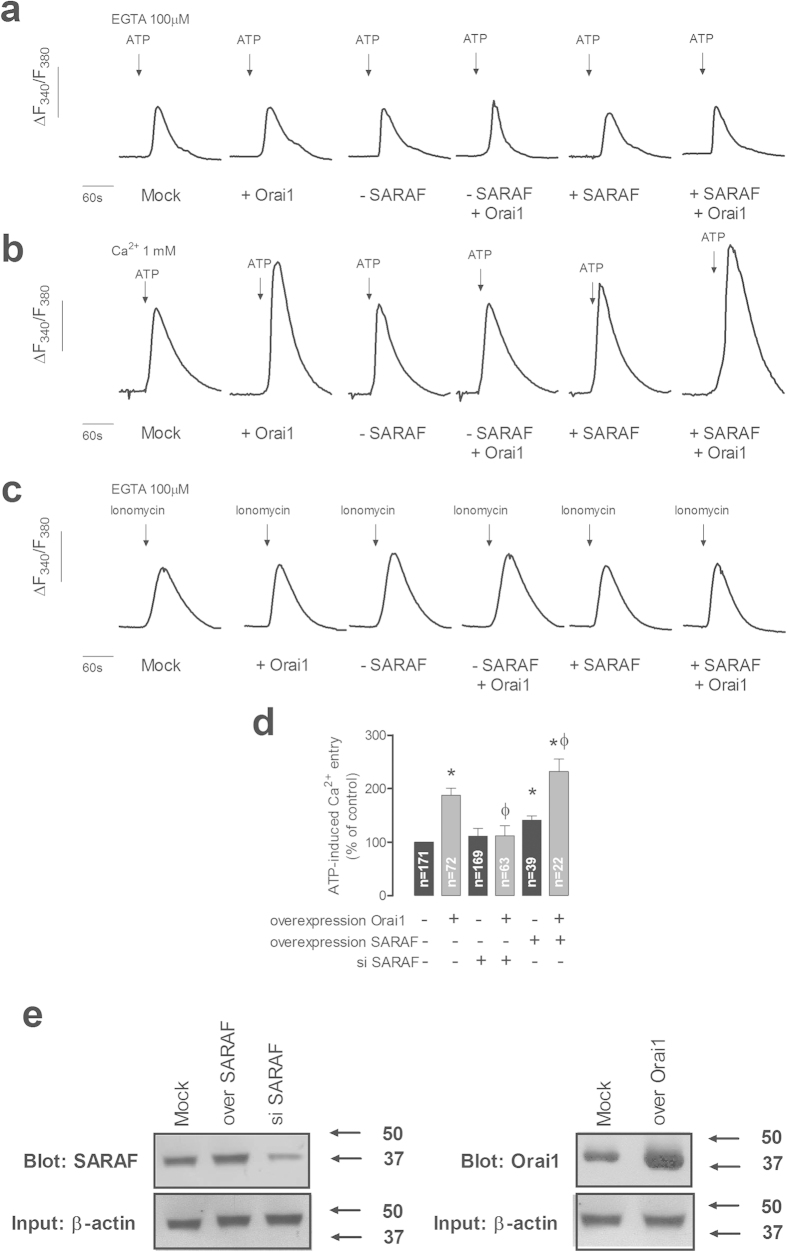
SARAF regulates Orai1 channel function. (**a–c**) NG115-401L cells were transfected with Orai1, SARAF, siSARAF or empty vector as control (Mock). Cells were perfused with a Ca^2+^-free medium (100 μM EGTA added) (**a,c**) or a medium containing 1 mM Ca^2+^ (**b**) and then stimulated with ATP (100 μM; (**a,b)**) or ionomycin (2 μM, (**c)**). Data are original traces representative of 22–171 experiments. (**d**) The bar graph represents ATP-evoked Ca^2+^ entry estimated as the integral of the rise in [Ca^2+^]_c_ above basal for 1½ min after the addition of ATP in the presence of external Ca^2+^, corrected by subtraction of the integral over the same period for stimulation with ATP in the absence of external Ca^2+^. Data are expressed as mean ± SEM and presented as percentage of control (mock-treated cells). * and ^ϕ^ represent p < 0.05 as compared to ATP-induced Ca^2+^ entry in mock-treated controls or cells overexpressing Orai1, respectively. (**e**) Immunoblot analysis of SARAF and Orai1 expressed in NG115-401L cells before or after treatment with siSARAF or expression plasmids for SARAF and Orai1 using anti-SARAF antibody and anti-Orai1 antibody (aa 288–301 (Sigma)). Membranes were reprobed with anti-β-actin antibody for protein loading control. The panel shows results from one experiment representative of 3 others. Molecular masses indicated on the right were determined using molecular-mass markers run in the same gel.

To understand the functional significance of the interaction of SARAF with Orai1 independently of STIM1 we determined Ca^2+^ entry in NG115-401L cells transfected with expression plasmid for Orai1 in combination with SARAF overexpression or siSARAF. Since NG115-401L cells release Ca^2+^ in response to TG but do not display SOCE^21^, we used ATP, as a physiological agonist, to stimulate Ca^2+^ entry. As depicted in Fig. 5a, stimulation of NG115-401L cells with 100 μM ATP in a Ca^2+^-free medium resulted in a transient rise in [Ca^2+^]_c_ due to Ca^2+^ release from the intracellular stores. Overexpression of Orai1 alone or in combination with either SARAF silencing or overexpression did not significantly modify the ability of ATP to release Ca^2+^ from the stores. If we consider the entry of Ca^2+^ stimulated by ATP, Orai1 overexpression resulted in a significant increase in Ca^2+^ influx to 187 ± 12% of control (Fig. 5b; p < 0.05), thus suggesting functional expression of Orai1. SARAF silencing did not significantly altered ATP-induced Ca^2+^ entry per se but abolished the additional Ca^2+^ influx observed in cells overexpressing Orai1 (Fig. 5b,d; p < 0.05). By contrast, SARAF overexpression significantly increased ATP-stimulated Ca^2+^ influx in the wild type as well as in the Orai1-overexpressing NG115-401L cells (Fig. 5b,d; p < 0.05). Figure. 5c shows the amount of Ca^2+^ accumulated in the intracellular Ca^2+^ stores, estimated by organelle content release using ionomycin (2 μM). As depicted in Fig. 5c, Orai1 expressing did not significantly alter the ability of NG115-401L cells to accumulate Ca^2+^ into the stores but SARAF silencing significantly enhanced the amount of Ca^2+^ released using ionomycin by 20% (p < 0.05) as previously described in HeLa and Jurkat cells^18^. Figure 5e, shows the expression of SARAF and Orai1 proteins in cells treated as mentioned above. These findings indicate that SARAF plays a positive regulatory role on Orai1 channel function, and, together with the data presented in Figs 2, 3, 4, provide evidence for the first time of a direct and functional interaction of SARAF with Orai1.

### Blockade of the C-terminal region of Orai1 displays a significant reduction in SARAF binding

**Figure 6:**
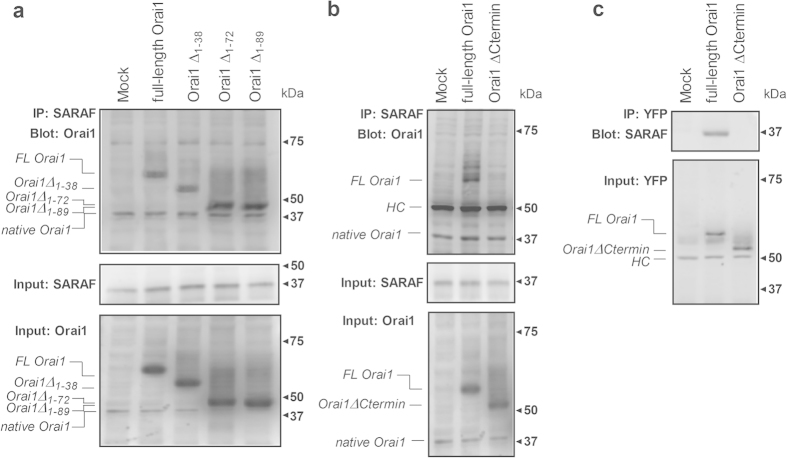
Analysis of the Orai1 site that interacts with SARAF. NG115-401L cells were transfected with SARAF alone or in combination with either pEYFP-full-length Orai1 (*FL-Orai1*), the N-terminal deletion mutants (pEYFP-Orai1 ΔN_1–38_ (*Orai1* Δ*N*_*1–38*_), ΔN_1–72_ (*Orai1* Δ*N*_*1–72*_) and ΔN_1–89_ (*Orai1* Δ*N*_*1–89*_) (**a**), pEYFP-Orai1 C-terminal deletion mutant (*Orai1*-Δ*Ctermin*, amino acids 1–260 (**b**,**c**) or empty vector (Mock), as indicated. After 48 h cells were lysed and the whole cell lysates were immunoprecipitated (IP) with anti-SARAF antibody (**a**,**b**) or anti-YFP antibody (**c**). Immunoprecipitates were subjected to 10% SDS-PAGE and subsequent Western blotting with a specific anti-Orai1 (amino acids 288–301, Sigma) antibody (**a**), anti-Orai1 (ab177021, Abcam) antibody (**b**) or anti-SARAF antibody (**c**). Membranes were reprobed with the antibody used for immunoprecipitation for protein loading control (middle panels). (**a**,**b**) Alternatively, the cell lysates were subjected to 10% SDS-PAGE and subsequent Western blotting with anti-Orai1 antibody (specific for amino acids 288–301; panel a, bottom) or with anti-Orai1 (ab177021, Abcam; panel b, bottom). HC, heavy chain of the antibody used for immunoprecipitation. The panels show results from one experiment representative of 3 others. Molecular masses indicated on the right were determined using molecular-mass markers run in the same gel.

Finally, we have investigated the location of the possible SARAF binding site of Orai1 in NG115-401L cells overexpressing pEYFP-Orai1 (full-length), the N-terminal deletion mutants (pEYFP-Orai1 ΔN_1–38_, ΔN_1–72_ and ΔN_1–89_) or the C-terminal deletion mutant (Orai1-ΔCtermin, amino acids 1–260) together with SARAF. As shown in Fig. 6a, our results indicate that co-expression of pEYFP-Orai1 and SARAF show detectable co-immunoprecipitation between both proteins, which was not altered in cells co-transfected with SARAF and the N-terminal truncations of Orai1 described above, which strongly indicates that the cytosolic N-terminal region of Orai1 is not required for SARAF interaction. By contrast, we were unable to detect co-immunoprecipitation between SARAF and the Orai1-ΔCtermin (Fig. 6b,c), thus suggesting that SARAF interacts with the C-terminus of Orai1.

To further assess the relevance of the C-terminal region of Orai1 in the interaction with SARAF we used a manoeuvre based on the blockade of this region by introduction of an antibody that specifically recognizes the sequence 288–301 of Orai1 (anti-Orai1CT, Sigma) as compared to the introduction of an antibody that recognizes 18 amino acids located in the N-terminal region (anti-Orai1NT, Prosci). The presence of these antibodies inside the cells was investigated by immunoprecipitation without adding any additional anti-Orai1 antibody and subsequent Western blotting with the anti-Orai1 antibody (288–301, Sigma)^22^. As shown in the supplemental Fig. 2a, first lane, Orai1 was clearly detected in cells that had been previously transfected with anti-Orai1CT or anti-Orai1NT antibodies. A faint band was detected when the Western blot was performed in the absence of primary antibody (primary-free control; supplemental Fig. 2a second lane), which reveals that the band detected by Western blotting using anti-Orai1 antibody was specifically Orai1. Cells transfection with anti-Orai1CT significantly attenuated the interaction of Orai1 with SARAF as compared to cells transfected with anti-Orai1NT or non-specific rabbit IgG (supplemental Fig. 2b,c). The lack of effect of transfection with anti-Orai1NT confirms the results obtained using different N-terminal deletion mutants. These findings indicate that SARAF interacts with a site located in the C-terminus of Orai1.

## Discussion

The present study provides relevant information concerning the mechanism of SOCE regulation by SARAF. We have found for the first time that SARAF associates with Orai1 and enhances Orai1 function in STIM1-deficient cells. SARAF interacts with the C-terminal region of Orai1 since Orai1 constructs lacking the C-terminus are unable to associate with SARAF and blockade of this region also impairs its ability to interact with SARAF. Our results suggest that SARAF dissociates from STIM1 upon Ca^2+^ store discharge probably to interact with Orai1 and cooperate with STIM1 in the activation of Orai1 and, then SARAF rapidly re-associates with STIM1 (and dissociates from Orai1) to participate in the inactivation of SOCE. Our results indicate that SARAF interacts and activates Orai1 channels in a scenario that allows direct association of both proteins independently of STIM1, which has been reported to be negative regulated by SARAF^17^,^18^.

Current evidence indicates that the Orai1 forming channels (i.e. CRAC, arachidonic acid-regulated Ca^2+^ and leukotriene C4-regulated Ca^2+^ channels^23^,^24^,^25^) are mostly regulated by STIM1. Similarly, there is a body of evidence for a role of STIM1 in the regulation of TRP channels as components of store-operated Ca^2+^ (SOC) channels, also involving Orai1 subunits^22^,^26^,^27^,^28^,^29^,^30^, Interestingly, Jha and coworkers have reported that, while in the resting state SARAF binds to SOAR in STIM1 (a process regulated by the CTID lobes), activation of Orai1 after Ca^2+^ store depletion requires dissociation of SARAF to allow SOAR to interact with and activate Orai1 channels. Subsequently, SARAF associates again with STIM1 to mediate SCDI. These findings are based on the observation that Orai1 current activated by the constitutively active STIM1(4E/4A) and STIM1(D76A) mutants was unaffected by SARAF, as well as on the role of different STIM1 mutants on SCDI of Orai1 currents^17^. In agreement with this study, we have found that SARAF, which co-immunoprecipitates with STIM1 under resting conditions, dissociates from STIM1 upon Ca^2+^ store depletion reaching a minimum after 30s of stimulation and then reinteracts with STIM1, and opposite results were observed when we tested the interaction of SARAF with Orai1, revealing that 30s after the initiation of Ca^2+^ store depletion there is an increase in SARAF-Orai1 co-immunoprecipitation followed by a decrease in the association between both proteins. Using STIM1-deficient NG115-401L cells we have found that dissociation of SARAF and Orai1 observed 30 s after treatment with TG occurs independently of STIM1. The dynamic interactions of SARAF with STIM1 or Orai1 were found to occur with the same time courses, which might suggest that SARAF dissociates from STIM1 in the initial stages of Ca^2+^ store depletion to interact with Orai1, although the mechanism underlying this translocation is unclear at this stage.

The interaction of SARAF with Orai1 might transiently cooperate with STIM1 in the activation of Orai1 currents. However, the predominant role of SARAF on SOCE is the negative regulation of Ca^2+^ entry via activation of SCDI as evidenced by us in manoeuvres modifying the expression of SARAF in MEG01 cells endogenously expressing STIM1 and Orai1 or by other authors in different cell types^17^,^18^,^31^, where SARAF overexpression or silencing SARAF expression leads to attenuated or enhanced SOCE, respectively. The lack of observation of a positive role of SARAF on the initial stages of SOCE suggests that the transient stimulatory role of SARAF on the activation of Orai1 might be a redundant function in the presence of functional STIM1, which might mask this effect and evidence solely the long lasting negative regulatory role of SARAF underlying the activation of SCDI. Alternatively, the lack of detection of a transient positive effect of SARAF overexpression in the initial stages of SOCE might be attributed to a negative regulation of other STIM1-regulated channels involved in SOCE by SARAF, such as TRPC proteins^23^,^32^,^33^,^34^. Still remains to be resolved what is the functional role of SARAF in cells with a low expression of STIM1 but with a significant expression of Orai1 and other Ca^2+^ permeable channels. Our results indicate that, in cells with a low expression of STIM1, SARAF enhances Orai1 function, which might play a positive role in Ca^2+^ entry through store-independent mechanisms. Unveiling the role of SARAF on TRP channel function in cells with a low STIM1 expression might provide relevant information concerning the role of SARAF on store-independent Ca^2+^ entry.

In summary, on the base of the previous studies^17^,^18^ and our findings, we propose a model where SARAF modulates Orai1 channel function through direct and indirect interaction. At rest, SARAF is associated with the SOAR/OASF region of STIM1 via the CTID domain; however, upon Ca^2+^ store depletion SARAF transiently dissociates from STIM1 and associates with the C-terminal region of Orai1. A few seconds later, and probably mediated by the increase in [Ca^2+^]_c_, SARAF dissociates from Orai1 and reinteracts with STIM1. Dissociation of SARAF from Orai1, together with the subsequent association with STIM1 might have a role in the SCDI of Orai1.

## Methods

### Materials

Fura-2 acetoxymethyl ester (fura-2/AM), was from Molecular Probes (Leiden, The Netherlands). Thapsigargin (TG), ATP, rabbit polyclonal anti-Orai1 antibody (catalog number O8264, epitope: amino acids 288–301 of human Orai1), Duolink^®^ *In Situ* Red Starter Kit Mouse/Rabbit, mouse monoclonal anti-Orai1 antibody (clone ORAI1-89, epitope: synthetic peptide located near the C-terminus of human Orai1, catalog number: SAB4200273), rabbit polyclonal anti-β-actin antibody (catalog number A2066, epitope: amino acids 365–375 of human β-actin), and bovine serum albumin (BSA) were from Sigma (Madrid, Spain). Rabbit polyclonal anti-TMEM66 (SARAF) antibody (catalog number PA5-24237. epitope: amino acids 33–62 of the N-terminal region of human TMEM66) and Turbofect transfection reagent were from Thermo Fisher (Madrid, Spain). Mouse monoclonal anti STIM1 antibody (Clone 44/GOK, epitope: amino acids 25–139 of human STIM1, catalog number 610954) was from BD Transduction Laboratories (Frankin Lakes, NJ, U.S.A.). Calmidazolium chloride was from Bio-Rad (Madrid, Spain). Rabbit polyclonal anti-Orai1 antibody (catalog number: ab177021, epitope: peptide from the extracellular loop 2 region), horseradish peroxidase-conjugated anti-mouse IgG antibody and anti-rabbit IgG antibody for IP (not recognizing the heavy and light chains of the immunoprecipitating antibody) were from Abcam (Madrid, Spain). Rabbit polyclonal anti-Orai1 antibody (catalog number: 4041, epitope: raised against an 18 amino acid synthetic peptide from near the N-terminus of human Orai1) was from Prosci (Fort Collins, CO, USA). Amaxa nucleofector kit C was purchased from (Lonza, Madrid, Spain). Protein A-agarose was from Upstate Biotechnology Inc. (Madrid, Spain). Mouse anti-GFP antibody (mixture of two monoclonal antibodies, clones 7.1 and 13.1, which recognize both wild-type and mutant forms of GFP, such as YFP^35^; catalogue number 11814460001) and complete EDTA-free protease inhibitor tablets were from Roche (Madrid, Spain). Enhanced chemiluminescence detection reagents were from Pierce (Cheshire, U. K.). TransPass P was from New England Biolabs (Ipswich, MA, U.S.A.). Taq Polimerase, T4 ligase, EcoRI and EcoRV enzymes were from Takara (Bio Inc, Japan). Ultraclean GelSpin Kit^®^ and Ultraclean Midi plasmid prep Kit^®^ were from MoBio (MO BIO Laboratories, Carlsbad, USA). All other reagents were of analytical grade.

### Plasmids construction

Plasmids were based on the previously published SARAF sequences (GenBank: JQ348891.1). The DNA of the complete cds was isolated from NG115-401L cells using specific primers (Forward: 5′ AAAAAACCCGGGATGGCCGCAGCCTGCGGGCC 3′; and reverse: 5′- AAAAAAGAATTCTTATCGTCTCCTGGTACCACCATAT-3′). Final cDNA was purified and cloned into the EcoRV site previously inserted in the pIRES2-eGFP-RV expression vector. Nucleotide sequence of this construct was verified by sequencing.

To knockdown expression of SARAF1, a pLKO.1-puro plasmid–based shRNA targeting the sequence: CGGACTTAGATATTGCATACA (clone ID: TRCN0000146643; Sigma-Aldrich) was used (SARAF-shRNA). In addition, a non-targeting shRNA plasmid (NT-shRNA) that targets no known human sequence was used as a control. A primer containing the target sequence along with a stem loop followed by the reverse target sequence was annealed to a complimentary primer and inserted into the EcoRI and AgeI sites of the pLKO.1-puro plasmid (Addgene; number 10878). The resulting hairpin consisted of the following sequence: 5′-CCGGCGGACTTAGATATTGCATACACTCGAGTGTATGCAATATCTAAGTCCGTTTTTTG-3′. The correct insertion of the hairpin into pLKO.1 plasmid was finally checked by sequencing.

### Cell culture and transfection

The megakaryoblastic cell line MEG01 was used in this study as a cellular model endogenously expressing STIM1. Moreover, STIM1-deficient NG115-401L cells were used for certain experimental procedures to avoid interference with endogenous STIM1. MEG01 and NG115-401L cell lines were obtained from ATCC (Manassas, VA, USA) and Sigma (Madrid, Spain), respectively, and cultured at 37 °C with a 5% CO_2_ in RPMI or DMEM, respectively, supplemented with 10% (v/v) fetal bovine serum, 2 mM L-glutamine and 100 U/mL penicillin and streptomycin.

Cells were transfected with expression plasmids for pIRES2-EGFP-SARAF, pEYFP-Orai1, pEYPF-OASF, the pEYFP-Orai1 N-terminal deletion mutants (Orai1 ΔN_1–38_, ΔN_1–72_ and ΔN_1–89_) or the pEYFP-Orai1 C-terminal deletion mutant (Orai1-ΔCtermin, amino acids 1–260) kindly provided by Dr. Romanin, as well as with the siSARAF or scramble plasmid as described previously^36^,^37^,^38^ using Amaxa nucleofector technology (for MEG-01 cells) or Turbofect transfection reagent (for NG115-401L cells) and were used 48 h after transfection. For transfection with antibodies we used transpass P following the manufacturer’s instructions and were used 6h after transfection.

Cell transfection with shOrai1 was performed as described previously^39^. For the shOrai1, the sense sequence was 5′-CACCTCACTGGTTAGCCATAAGACGAATCTTATGGCTAACCAGTGA-3′, and the antisense sequence was 5′-AAAACCTTTACACGCTAGATGGTTTGCTCTTATGGCTAACCAGTGA-3′. Plasmids were used for silencing experiments at 1 μg/μl.

### Measurement of cytosolic free-calcium concentration ([Ca^2+^]_c_)

Cells were loaded with fura-2 by incubation with 2 μM fura 2/AM for 30 min at room temperature. Coverslips with cultured cells were mounted on a perfusion chamber and placed on the stage of an epifluorescence inverted microscope (Nikon Diaphot T200, Melville, NY, USA) with image acquisition and analysis system for videomicroscopy (Hamamatsu Photonics, Hamamatsu, Japan). Cells were continuously superfused with HEPES-buffered saline (HBS) containing (in mM): 125 NaCl, 5 KCl, 1 MgCl2, 5 glucose, 25 HEPES, and pH 7.3, supplemented with 0.1% (w/v) BSA.. Cells were alternatively excited with light from a xenon lamp passed through a high-speed monochromator (Polychrome IV, Photonics, Hamamatsu, Japan) at 340/380 nm. Fluorescence emission at 505 nm was detected using a cooled digital CCD camera (Hisca CCD C-6790, Hamamatsu, Japan) and recorded using Aquacosmos 2.5 software (Hamamatsu Photonics, Hamamatsu, Japan). Fluorescence ratio (F_340_/F_380_) was calculated pixel by pixel and data are presented as ΔF_340_/F_380_. ATP-evoked Ca^2+^ influx was measured as the integral of the rise in [Ca^2+^]_c_ above basal for 1½ min after the addition of ATP in the presence of external Ca^2+^, corrected by subtraction of the integral over the same period for stimulation in the absence of external Ca^2+^ (with 100 μM EGTA).

### Immunoprecipitation and Western blotting

The immunoprecipitation and Western blotting were performed as described previously^40^. Briefly, 500 μL aliquots of cell suspension (4 × 10^6 ^cell/mL) were lysed with an equal volume of ice-cold 2×NP-40 buffer, pH 8, containing 274 mM NaCl, 40 mM Tris, 4 mM EDTA, 20% glycerol, 2% nonidet P-40, 2 mM Na_3_VO_4_ and complete EDTA-free protease inhibitor tablets. Aliquots of cell lysates (1 mL) were immunoprecipitated by incubation with 1 μg of anti-STIM1 or 2 μg of anti-SARAF or anti-Orai1 antibody and 25 μL of protein A-agarose overnight at 4 °C on a rocking platform. The immunoprecipitates were resolved by 8% SDS-PAGE and separated proteins were electrophoretically transferred onto nitrocellulose membranes for subsequent probing. Blots were incubated overnight with 10% (w/v) BSA in tris-buffered saline with 0.1% Tween 20 (TBST) to block residual protein binding sites. Immunodetection of Orai1, STIM1 and SARAF was achieved by incubation for 2h with anti-STIM1 antibody diluted 1:250 in TBST, anti-Orai1 antibody diluted 1:200 in TBST, the anti-SARAF antibody diluted 1:1000 in TBST and anti-calmodulin antibody diluted 1:500. The primary antibody was removed and blots were washed six times for 5 min each with TBST. To detect the primary antibody, blots were incubated for 1 h with horseradish peroxidase-conjugated goat anti-mouse IgG antibody or horseradish peroxidase-conjugated goat anti-rabbit IgG antibody diluted 1:10000 in TBST and then exposed to enhanced chemiluminiscence reagents for 4 min. The density of bands was measured using C-DiGit Chemiluminescent Western Blot Scanner. Data were normalized to the amount of protein recovered by the antibody used for the immunoprecipitation.

### Proximity ligation assay

The PLA was performed using the Duolink^®^ *In Situ* Red Starter Kit Mouse/Rabbit and performed following the manufacturer’s instructions and as previously described^41^. After stimulation, the cells were fixed in ice-cold 4% paraformaldehyde in phosphate-buffered saline (PBS) for 10 min. The cells were then washed three times with PBS with agitation and permeabilized in PBS that contained 0.5% Triton X-100 for 10 min at room temperature, followed by washing the cells twice with 0.05% Tween 20 in Tris-buffered saline buffer (TBS, containing in mM: 50 Tris-HCl [pH 7.5] and 150 NaCl). Cells were then blocked for 30 min with one drop of Duolink Blocking solution in a humidified chamber at 37 °C and incubated for 1 h at 37 °C with appropriate combinations of antibodies diluted 1:100 in Duolink Antibody Diluent solution (40 μL). The antibodies used for the PLA were rabbit anti-TMEM66 (SARAF) (Thermo Fisher, Madrid, Spain) combined with mouse anti-Orai1 (Sigma, Madrid, Spain) or mouse anti-STIM1 (BD Transduction Laboratories, Frankin Lakes, NJ, U.S.A.). After washing with Wash Buffer A, the cells were incubated for 1 h at 37 °C with PLA probes, which are secondary antibodies (Duolink^®^ *In Situ* PLA Probe anti-rabbit PLUS or Duolink^®^ *In Situ* PLA Probe anti-mouse MINUS) conjugated to unique oligonucleotides. The cultures were further subjected to *in situ* PLA using a Duolink Detection kit according to the manufacturer’s instructions. Briefly, the slides were incubated with Ligation-Ligase solution in a pre-heated humidified chamber for 30 min at 37 °C, followed by incubation with amplification polymerase solution for an additional 100 min at 37 °C. Finally, the cells were washed in Wash Buffer B, and the slides were mounted using Mounting Medium with DAPI^®^ and evaluated using a Eclipse TE300 fluorescence microscope with a ×60 oil-immersion objective (Nikon Corporation, Tokyo, Japan). The quantification of PLA signals was performed using ImageJ software (NIH, U.S.A.). The processed images were thresholded and the *in situ* PLA signals per cell were quantified. The settings were kept constant for all of the images throughout the experiments. Quantifications were performed from 30 to 40 images from a minimum of four slides for each treatment corresponding to 100–200 cells. Mouse anti-Orai1 directed towards the C-terminus (catalog number SAB4200273, Sigma) combined with rabbit anti-Orai1 directed towards 18 amino acids located in the N-terminal region of human Orai1 (catalog number 4041, Prosci) was used as a positive control. As a negative technical control, the primary antibodies were used alone, or both primary antibodies were omitted. The negative controls did not yield any significant PLA signals at any treatment condition.

### Statistical Analysis

Analysis of statistical significance was performed using one-way analysis of variance. For comparison between two groups Student’s *t* test was used. *P* < 0.05 was considered to be significant for a difference.

## Additional Information

**How to cite this article**: Albarran, L. *et al*. Dynamic interaction of SARAF with STIM1 and Orai1 to modulate store-operated calcium entry. *Sci. Rep*. **6**, 24452; doi: 10.1038/srep24452 (2016).

## Supplementary Material

Supplementary Information

## References

[b1] Roos J. . STIM1, an essential and conserved component of store-operated Ca^2+^ channel function. J Cell Biol 169, 435–445 (2005). 15866891 10.1083/jcb.200502019 PMC2171946

[b2] Liou J. . STIM is a Ca^2+^ sensor essential for Ca^2+^-store-depletion-triggered Ca^2+^ influx. Curr Biol 15, 1235–1241 (2005). 16005298 10.1016/j.cub.2005.05.055 PMC3186072

[b3] Feske S. . A mutation in Orai1 causes immune deficiency by abrogating CRAC channel function. Nature 441, 179–185 (2006). 16582901 10.1038/nature04702

[b4] Vig M. . CRACM1 multimers form the ion-selective pore of the CRAC channel. Curr Biol 16, 2073–2079 (2006). 16978865 10.1016/j.cub.2006.08.085 PMC5685803

[b5] Zhang S. L. . Genome-wide RNAi screen of Ca^2+^ influx identifies genes that regulate Ca^2+^ release-activated Ca^2+^ channel activity. Proc Natl Acad Sci USA 103, 9357–9362 (2006). 16751269 10.1073/pnas.0603161103 PMC1482614

[b6] Hoth M. & Penner R. Calcium release-activated calcium current in rat mast cells. J Physiol 465, 359–386 (1993). 8229840 10.1113/jphysiol.1993.sp019681 PMC1175434

[b7] Derler I. . A Ca^2+^ Release-activated Ca^2+^ (CRAC) Modulatory Domain (CMD) within STIM1 Mediates Fast Ca^2+^-dependent Inactivation of ORAI1 Channels. J Biol Chem 284, 24933–24938 (2009). 19622747 10.1074/jbc.C109.024083 PMC2757196

[b8] Zweifach A. & Lewis R. S. Slow calcium-dependent inactivation of depletion-activated calcium current. Store-dependent and -independent mechanisms. J Biol Chem 270, 14445–14451 (1995). 7540169 10.1074/jbc.270.24.14445

[b9] Parekh A. B. Slow feedback inhibition of calcium release-activated calcium current by calcium entry. J Biol Chem 273, 14925–14932 (1998). 9614097 10.1074/jbc.273.24.14925

[b10] Stathopulos P. B., Zheng L., Li G. Y., Plevin M. J. & Ikura M. Structural and mechanistic insights into STIM1-mediated initiation of store-operated calcium entry. Cell 135, 110–122 (2008). 18854159 10.1016/j.cell.2008.08.006

[b11] Yuan J. P., Zeng W., Dorwart M. R., Choi Y. J., Worley P. F. & Muallem S. SOAR and the polybasic STIM1 domains gate and regulate Orai channels. Nat Cell Biol 11, 337–343 (2009). 19182790 10.1038/ncb1842 PMC2663385

[b12] Muik M. . A Cytosolic Homomerization and a Modulatory Domain within STIM1 C Terminus Determine Coupling to ORAI1 Channels. J Biol Chem 284, 8421–8426 (2009). 19189966 10.1074/jbc.C800229200 PMC2659200

[b13] Park C. Y. . STIM1 clusters and activates CRAC channels via direct binding of a cytosolic domain to Orai1. Cell 136, 876–890 (2009). 19249086 10.1016/j.cell.2009.02.014 PMC2670439

[b14] Kawasaki T., Lange I. & Feske S. A minimal regulatory domain in the C terminus of STIM1 binds to and activates ORAI1 CRAC channels. Biochem Biophys Res Commun 385, 49–54 (2009). 19433061 10.1016/j.bbrc.2009.05.020 PMC2821023

[b15] Muik M. . STIM1 couples to ORAI1 via an intramolecular transition into an extended conformation. EMBO J 30, 1678–1689 (2011). 21427704 10.1038/emboj.2011.79 PMC3101990

[b16] Yang X., Jin H., Cai X., Li S. & Shen Y. Structural and mechanistic insights into the activation of Stromal interaction molecule 1 (STIM1). Proc Natl Acad Sci USA 109, 5657–5662 (2012). 22451904 10.1073/pnas.1118947109 PMC3326449

[b17] Jha A. . The STIM1 CTID domain determines access of SARAF to SOAR to regulate Orai1 channel function. J Cell Biol 202, 71–79 (2013). 23816623 10.1083/jcb.201301148 PMC3704993

[b18] Palty R., Raveh A., Kaminsky I., Meller R. & Reuveny E. SARAF inactivates the store operated calcium entry machinery to prevent excess calcium refilling. Cell 149, 425–438 (2012). 22464749 10.1016/j.cell.2012.01.055

[b19] Albarran L., Lopez J. J., Dionisio N., Smani T., Salido G. M. & Rosado J. A. Transient receptor potential ankyrin-1 (TRPA1) modulates store-operated Ca^2+^ entry by regulation of STIM1-Orai1 association. Biochim Biophys Acta 1833, 3025–3034 (2013). 23994313 10.1016/j.bbamcr.2013.08.014

[b20] Csutora P. . Novel role for STIM1 as a trigger for calcium influx factor production. J Biol Chem 283, 14524–14531 (2008). 18337241 10.1074/jbc.M709575200 PMC2386937

[b21] Albarran L. . TRPC6 participates in the regulation of cytosolic basal calcium concentration in murine resting platelets. Biochim Biophys Acta 1843, 789–796 (2014). 24462772 10.1016/j.bbamcr.2014.01.014

[b22] Lopez J. J., Salido G. M., Pariente J. A. & Rosado J. A. Interaction of STIM1 with endogenously expressed human canonical TRP1 upon depletion of intracellular Ca^2+^ stores. J Biol Chem 281, 28254–28264 (2006). 16870612 10.1074/jbc.M604272200

[b23] Huang G. N. . STIM1 carboxyl-terminus activates native SOC, I(crac) and TRPC1 channels. Nat Cell Biol 8, 1003–1010 (2006). 16906149 10.1038/ncb1454

[b24] Shuttleworth T. J., Thompson J. L. & Mignen O. STIM1 and the noncapacitative ARC channels. Cell Calcium 42, 183–191 (2007). 17391754 10.1016/j.ceca.2007.01.012 PMC1995027

[b25] Zhang X. . Complex role of STIM1 in the activation of store-independent Orai1/3 channels. J Gen Physiol 143, 345–359 (2014). 24567509 10.1085/jgp.201311084 PMC3933941

[b26] Yuan J. P. . TRPC channels as STIM1-regulated SOCs. Channels (Austin) 3, 221–225 (2009). 19574740 10.4161/chan.3.4.9198

[b27] Zeng W. . STIM1 gates TRPC channels, but not Orai1, by electrostatic interaction. Mol Cell 32, 439–448 (2008). 18995841 10.1016/j.molcel.2008.09.020 PMC2586614

[b28] Cheng K. T., Liu X., Ong H. L. & Ambudkar I. S. Functional requirement for Orai1 in store-operated TRPC1-STIM1 channels. J Biol Chem 283, 12935–12940 (2008). 18326500 10.1074/jbc.C800008200 PMC2442339

[b29] Ong H. L. . Dynamic assembly of TRPC1-STIM1-Orai1 ternary complex is involved in store-operated calcium influx. Evidence for similarities in store-operated and calcium release-activated calcium channel components. J Biol Chem 282, 9105–9116 (2007). 17224452 10.1074/jbc.M608942200 PMC3309402

[b30] Jardin I., Lopez J. J., Salido G. M. & Rosado J. A. Orai1 mediates the interaction between STIM1 and hTRPC1 and regulates the mode of activation of hTRPC1-forming Ca^2+^ channels. J Biol Chem 283, 25296–25304 (2008). 18644792 10.1074/jbc.M802904200

[b31] Maleth J., Choi S., Muallem S. & Ahuja M. Translocation between PI(4,5)P2-poor and PI(4,5)P2-rich microdomains during store depletion determines STIM1 conformation and Orai1 gating. Nat Commun 5, 5843 (2014). 25517631 10.1038/ncomms6843 PMC4270102

[b32] Pani B. . Impairment of TRPC1-STIM1 channel assembly and AQP5 translocation compromise agonist-stimulated fluid secretion in mice lacking caveolin1. J Cell Sci 126, 667–675 (2013). 23203809 10.1242/jcs.118943 PMC3613184

[b33] Ng L. C. . TRPC1 and Orai1 interact with STIM1 and mediate capacitative Ca^2+^ entry caused by acute hypoxia in mouse pulmonary arterial smooth muscle cells. Am J Physiol Cell Physiol 303, C1156–1172 (2012). 23034388 10.1152/ajpcell.00065.2012

[b34] Jardin I., Salido G. M. & Rosado J. A. Role of lipid rafts in the interaction between hTRPC1, Orai1 and STIM1. Channels (Austin) 2, 401–403 (2008). 18843204 10.4161/chan.2.6.7055

[b35] Derler I. . Cholesterol modulates Orai1 channel function. Sci Signal 9, ra10 (2016). 26814231 10.1126/scisignal.aad7808 PMC5373433

[b36] Lopez E., Berna-Erro A., Salido G. M., Rosado J. A. & Redondo P. C. FKBP52 is involved in the regulation of SOCE channels in the human platelets and MEG 01 cells. Biochim Biophys Acta 1833, 652–662 (2013). 23228564 10.1016/j.bbamcr.2012.11.029

[b37] Dionisio N., Smani T., Woodard G. E., Castellano A., Salido G. M. & Rosado J. A. Homer proteins mediate the interaction between STIM1 and Cav1.2 channels. Biochim Biophys Acta 1853, 1145–1153 (2015). 25712868 10.1016/j.bbamcr.2015.02.014

[b38] Albarran L., Lopez J. J., Woodard G. E., Salido G. M. & Rosado J. A. Store-operated Ca^2+^ entry-associated regulatory factor (SARAF) plays an important role in the regulation of arachidonate-regulated Ca^2+^ (ARC) channels. J Biol Chem 291, 6982–6988 (2016). 26817842 10.1074/jbc.M115.704940 PMC4807282

[b39] Woodard G. E., Lopez J. J., Jardin I., Salido G. M. & Rosado J. A. TRPC3 regulates agonist-stimulated Ca^2+^ mobilization by mediating the interaction between type I inositol 1,4,5-trisphosphate receptor, RACK1, and Orai1. J Biol Chem 285, 8045–8053 (2010). 20022948 10.1074/jbc.M109.033605 PMC2832955

[b40] Redondo P. C., Ben-Amor N., Salido G. M., Bartegi A., Pariente J. A. & Rosado J. A. Ca^2+^-independent activation of Bruton’s tyrosine kinase is required for store-mediated Ca^2+^ entry in human platelets. Cell Signal 17, 1011–1021 (2005). 15894173 10.1016/j.cellsig.2004.11.019

[b41] Gruszczynska-Biegala J. & Kuznicki J. Native STIM2 and ORAI1 proteins form a calcium-sensitive and thapsigargin-insensitive complex in cortical neurons. J Neurochem 126, 727–738 (2013). 23711249 10.1111/jnc.12320

