# Supplementary material for: Dynamic interaction of SARAF with STIM1 and Orai1 to modulate store-operated calcium entry
Source: Sci Rep. 2016 Apr 12;6:24452. doi: 10.1038/srep24452 (PMC4828706; doi:10.1038/srep24452)
Supplement: Supplementary Information [file srep24452-s1.pdf]

SUPPLEMENTARY INFORMATION

Dynamic interaction of SARAF with STIM1 and Orai1 to modulate store-operated calcium entry

Letizia Albarran, Jose J. Lopez, Nidhal Ben Amor, Francisco E. Martin-Cano, Alejandro Berna-Erro, Tarik Smani, Gines M. Salido & Juan A. Rosado

Department of Physiology (Cellular Physiology and Muscle Physiology Research Groups), University of Extremadura, 10003 Caceres, and Department of Medical Physiology and Biophysic, Institute of Biomedicine of Sevilla, Sevilla, Spain

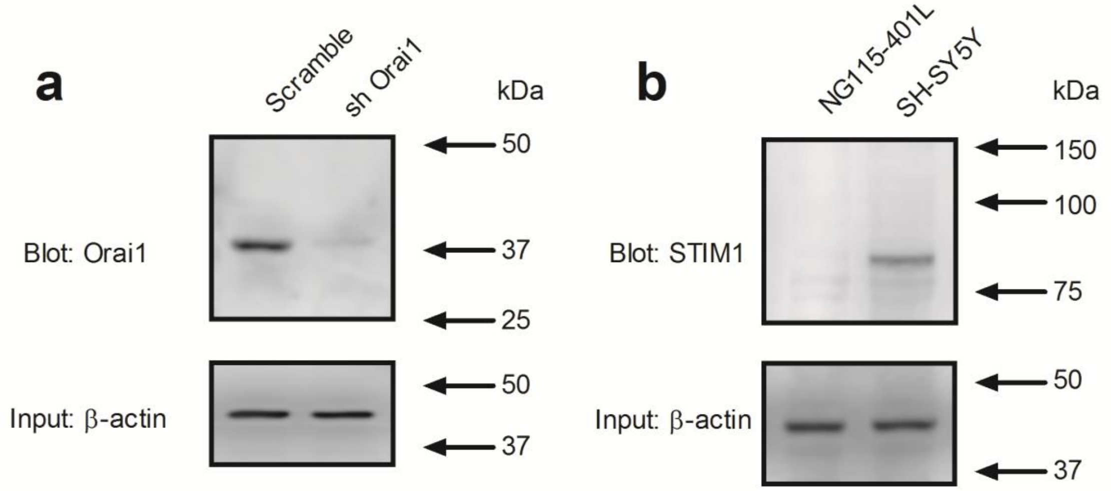

Supplemental Figure 1. Analysis of the specificity of Orai1 and STIM1 antibodies.

(**a**) NG115-401L cells were transfected either with shOrai1 or scramble plasmid. 48 h later cells were lysed and whole cell lysates were subjected to Western blotting using the anti-Orai1 (aa 288-301 (Sigma)) antibody or the anti-Orai1 (aa 22-40 (Abcam)) antibody, as indicated. Membranes were reprobed with anti-β actin antibody for protein loading control. (**b**) NG115-401L cells transfected with STIM1 overexpression plasmid or empty vector. 48 h later cells were lysed and whole cell lysates were subjected to Western blotting with anti-STIM1 antibody (BD Transduction Laboratories). Membranes were reprobed with anti-β actin antibody for protein loading control. The panels show results from one experiment representative of 3 others. Molecular masses indicated on the right were determined using molecular-mass markers run in the same gel.

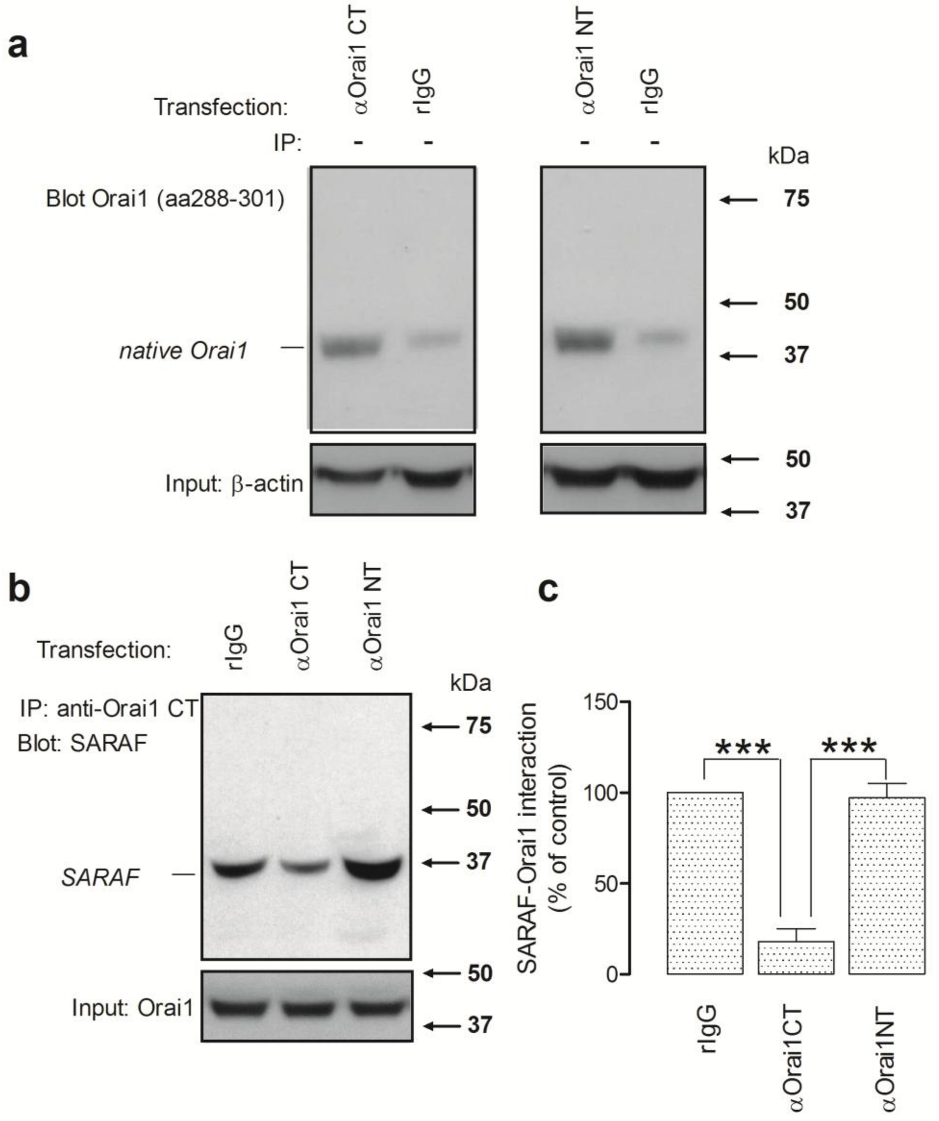

Supplemental Figure 2. SARAF interacts with the C-terminus of Orai1.

(**a**) NG115-401L cells were transfected either with anti-Orai1 antibody directed towards the sequence 288-301 (αOrai1CT (Sigma, Madrid, Spain)), with anti-Orai1 antibody directed towards 18 aminoacids located in the N-terminus (αOrai1NT, Prosci (Fort Collins, CO, USA)) or non-specific rabbit IgG (rIgG). Six hours later cells were lysed and whole cell lysates were immunoprecipitated with protein A-agarose in the absence of further antibodies and probed by Western blotting using the αOrai1CT (aa 288-301) antibody 1:200 in TBST. Membranes were reprobed with anti-β actin antibody for protein loading control. (**b**) cells transfected with rIgG, αOrai1CT or αOrai1NT. Six hours later cells were lysed and whole cell lysates were immunoprecipitated with αOrai1CT antibody. Immunoprecipitates were subjected to 10% SDS-PAGE and subsequent Western blotting with a specific anti-SARAF antibody. Membranes were reprobed with the immunoprecipitating antibody for protein loading control. The panels show results from one experiment representative of 3 others. Molecular masses indicated on the right were determined using molecular-mass markers run in the same gel. (**c**) The bar graph represents the quantification of SARAF-Orai1 association. Results are recorded as arbitrary optical density units, expressed as mean ± S.E.M. and presented as percentage of the interaction observed in cells transfected with rabbit IgG. *** represents $p < 0.001$ as compared to cells transfected with rabbit IgG or αOrai1NT.
